# Effect of Plant Growth-Promoting Bacteria on Antioxidant Status, Acetolactate Synthase Activity, and Growth of Common Wheat and Canola Exposed to Metsulfuron-Methyl

**DOI:** 10.3390/jox14010005

**Published:** 2024-01-02

**Authors:** Margarita Bakaeva, Sergey Chetverikov, Sergey Starikov, Aliya Kendjieva, Gaisar Khudaygulov, Darya Chetverikova

**Affiliations:** Ufa Institute of Biology, Ufa Federal Research Centre, Russian Academy of Sciences, 450054 Ufa, Russia; che-kov@mail.ru (S.C.); senik0406@gmail.com (S.S.); aliya_kendzieva@mail.ru (A.K.); bio.logos@yandex.com (G.K.); belka-strelka8031@yandex.ru (D.C.)

**Keywords:** acetolactate synthase, antioxidants, antioxidant enzymes, bacteria, biostimulant, herbicide, metsulfuron-methyl, safeners

## Abstract

Metsulfuron-methyl, a widely used herbicide, could cause damage to the sensitive plants in crop-rotation systems at extremely low levels in the soil. The potential of plant growth-promoting bacteria (PGPB) for enhancing the resistance of plants against herbicide stress has been discovered recently. Therefore, it is poorly understood how physiological processes occur in plants, while PGPB reduce the phytotoxicity of herbicides for agricultural crops. In greenhouse studies, the effect of strains *Pseudomonas protegens* DA1.2 and *Pseudomonas chlororaphis* 4CH on oxidative damage, acetolactate synthase (ALS), enzymatic and non-enzymatic antioxidants in canola (*Brassica napus* L.), and wheat (*Triticum aestivum* L.) were investigated under two levels (0.05 and 0.25 mg∙kg^−1^) of metsulfuron-methyl using spectrophotometric assays. The inoculation of herbicide-exposed wheat with bacteria significantly increased the shoots fresh weight (24–28%), amount of glutathione GSH (60–73%), and flavonoids (5–14%), as well as activity of ascorbate peroxidase (129–140%), superoxide dismutase SOD (35–49%), and ALS (50–57%). Bacterial treatment stimulated the activity of SOD (37–94%), ALS (65–73%), glutathione reductase (19–20%), and the accumulation of GSH (61–261%), flavonoids (17–22%), and shoots weight (27–33%) in herbicide-exposed canola. Simultaneous inoculation prevented lipid peroxidation induced by metsulfuron-methyl in sensitive plants. Based on the findings, it is possible that the protective role of bacterial strains against metsulfuron-metil is linked to antioxidant system activation.

## 1. Introduction

Metsulfuron-methyl is one of the most widely used herbicides in fields around the world because of its low cost and broad spectrum for controlling weeds. However, its ill-considered use can bring not only benefits but also some problems. The persistence time of sulfonylureas in soil can vary significantly depending on soil pH, organic carbon contents, microbial biomass, and silt/clay fraction [1,2]. Therefore, it can be difficult to predict it in a specific field without chemical analysis. On the other hand, metsulfuron-methyl can be toxic to sensitive plant species in crop-rotation systems at extremely low levels in the soil [3,4,5]. This leads to the need for means that would protect sensitive crops from methsulfuron-methyl residues.

The use of plant growth-stimulating bacteria (PGPB) is attracting increasing attention from researchers [6,7,8,9]. This is due to the growing concern of consumers of agricultural products about the toxic effects of pesticides, as well as the great potential of rhizobacteria to solve various problems of agronomy. The use of bacteria as biofungicides, bioinsecticides, and plant growth stimulants is a well-studied scientific problem. This is largely due to understanding the mechanisms by which bacteria suppress phytopathogenic microorganisms [10], make nutrients available to plants [11], modulate phytohormones [12], and remove phytotoxic metabolites from the soils [13]. There are examples of success in protecting crops from stress factors, mainly drought, salinization, and heavy metals [14,15]. The use of bacteria to reduce herbicidal stress has been demonstrated relatively recently and is reflected so far in a small number of publications. According to Ahemad and Khan [16], quizalafop-p-ethyl and clodinafop herbicides had a negative impact on chickpea growth, while the plants inoculated with *Mesorhizobium* sp. MRC4 grew better. Motamedi et al. [17] described a positive effect of the native plant growth-promoting bacteria on plant biomass in the presence and absence of imazethapyr herbicide. The protective effect of strains of *Pseudomonas putida* [18], *P. protegens* DA1.2 [19], *P. chlororaphis* PAS18 [20], and *Azotobacter salinestris* KF470807 [21] was studied. In the described experiments, an improvement in the growth of agricultural plants and the reduction of herbicide stress manifestations after spraying with bacterial cultures were recorded. However, little attention is paid to the study of the ways in which PGPB mitigate herbicidal stress.

The herbicidal effect of sulfonylureas is associated with the inhibition of enzymatic activity of acetolactate synthase (ALS). Sulfonylurea herbicides also seem to have some effect on reactive oxygen species accumulation in treated plants, although oxidative stress is a secondary consequence of ALS inhibition [22]. For example, the exposure of wheat seedlings to metsulfuron-methyl provoked oxidative stress and reduced antioxidant enzyme activity and nitric oxide (NO) content [23]. One of the largest sources of reactive oxygen species in plant stress derives from damage to chloroplasts and interference with photosynthesis. In rapeseed treatment with sulfonylurea and other ALS-inhibiting compounds resulted in the destruction of chloroplasts and photosynthesis incapability [24]. In our work, we studied the effect of protective growth-stimulating bacteria on oxidative damage, enzymatic and non-enzymatic antioxidants in canola, and wheat plants treated with metsulfuron-methyl. We also measured the activity of acetolactate synthase to determine whether the beneficial effect of the bacterium correlates with the activity of this enzyme. Rapeseed and wheat were chosen to study the reaction to the bacterial treatment of crops that vary greatly in sensitivity to metsulfuron-methyl. Wheat as a monocotyledonous plant is relatively resistant to methsulfuron-methyl. Wheat fields are often treated with this herbicide to control weeds. Canola, like most dicotyledonous plants, is sensitive even to small doses of metsulfuron-methyl. Although canola is not treated with metsulfuron-methyl intentionally, it can be damaged by herbicide residues in the soil. 

## 2. Materials and Methods

### 2.1. Plant and Microorganisms

The plants used in this study were common wheat (*Triticum aestivum* L. cv. Saratovskaya № 55) and canola (*Brassica napus* L. cv. Kupol). Seeds were obtained from the Bashkir Research Institute of Agriculture of the Ufa Federal Research Centre of the Russian Academy of Sciences, Russia.

The DA1.2 strain of *Pseudomonas protegens*, Ramette et al., and the strain 4CH of *Pseudomonas chlororaphis*, Bergey et al., were isolated previously by the authors of the articles [25,26]. The bacterium DA1.2 was deposited in the All-Russian Collection of Microorganisms under the number VCM B-3542D. These bacterial strains synthesize indole-3-acetic acid, fixe atmospheric nitrogen, solubilize ‘unavailable’ forms of calcium-bound P, and bio-control phytopathogenic fungi (Table 1). Both bacteria retain their properties under the herbicide metsulfuron-methyl at a dose of up to 1 g per 1 kg of substrate. The bacterial cultures were previously grown in King B medium (glycerol—10 g∙L^−1^, peptone—5 g∙L^−1^, K_2_HPO_4_—5 g∙L^−1^, MgSO_4_—1.5 g∙L^−1^) in an orbital shaker (160 rpm) at 28 °C for 72 h. They were then diluted with water to obtain a plant treatment suspension containing 10^5^ CFU∙mL^−1^ of bacteria.

### 2.2. Experimental Details

**Figure 1:**
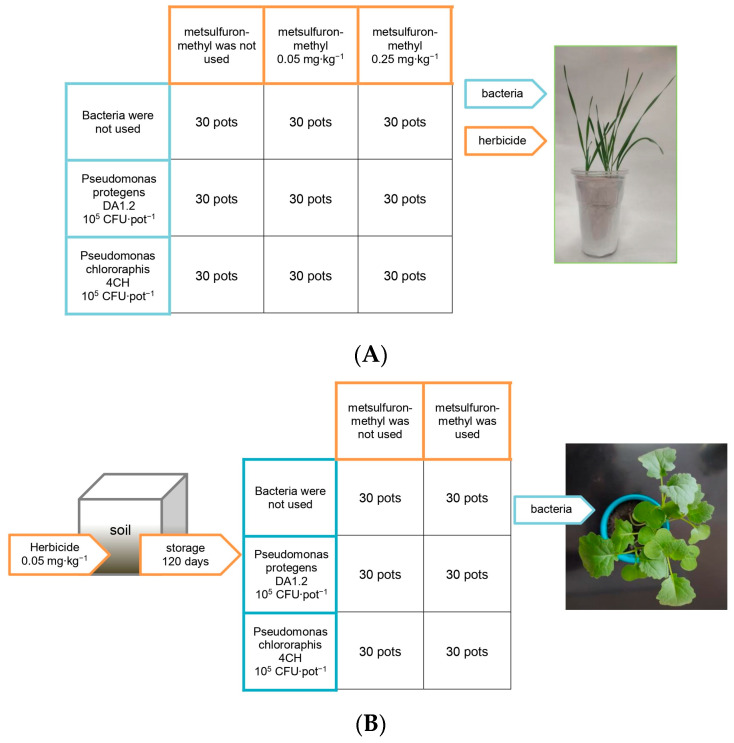
The scheme of wheat (**A**) and rapeseed (**B**) treatments. The figure does not indicate the location of the pots in the greenhouse.

The experiment was carried out in 2023 in the greenhouse of the Ufa Institute of Biology of the Ufa Federal Research Centre of the Russian Academy of Sciences (location: Russian Federation, Ufa, latitude 54.44, longitude 55.58, altitude 158 m). The soil was taken from the arable layer of Chernozem Haplic (C_org_ 3.5%, N_tot_ 0.45%, P_Egner_ 140 mg/kg, K_Egner_ 125 mg/kg, pH_KCl_ 6.5). Before the sampling, the soil had not been used in agriculture for three years. Herbicide metsulfuron-methyl was provided by LLC Pesticides ru (Russia). One week after planting, the wheat soil was treated with metsulfuron-methyl at doses of 0.05 mg∙kg^−1^ and 0.25 mg∙kg^−1^, corresponding to the recommended dose and its excess (Figure 1). This imitated the use of a herbicide for weed control in wheat crops. The soil intended for planting rapeseed was treated with metsulfuron-methyl at doses of 0.05 mg∙kg^−1^. Then, the treated soil was stored in ventilated containers for four months, maintaining a soil moisture content of 55 ± 5%. After that, the soil was used for the growing of canola. This simulated the effect on canola of herbicide residues previously used to protect other crops. 

Pre-germinated canola seeds were planted in 0.2 L pots, and pre-germinated wheat seeds were planted in 0.5 L pots, and both were grown under artificial lighting. The photon flux density was 190 μmol × m^−2^ × s^−1^; the photoperiod was 14 h; the temperature was 20–25 °C; and the soil moisture was 60 ± 5% of the water-holding capacity. After emergence, half of the seedlings were sprayed with a diluted bacterial culture. The other half were treated with a mixture of water and sterile King B nutrient medium at a ratio of medium:water of 1:10,000. Control plants were grown without exposure to herbicides or bacteria. Five seeds were used per pot, and thirty pots were used per experimental group. Thus, the plant groups were canola without treatments (control), canola exposed to bacteria DA1.2, canola exposed to bacteria 4CH, canola exposed to herbicide residues, canola exposed to bacteria DA1.2 and herbicide residues, canola exposed to bacteria 4CH and herbicide residues, wheat without treatments (control), wheat exposed to bacteria DA1.2, wheat exposed to bacteria 4CH, wheat exposed to herbicide 0.05 mg∙kg^−1^, wheat exposed to bacteria DA1.2 and herbicide 0.05 mg∙kg^−1^, wheat exposed to bacteria 4CH and herbicide 0.05 mg∙kg^−1^, wheat exposed to herbicide 0.25 mg∙kg^−1^, wheat exposed to bacteria DA1.2 and herbicide 0.25 mg∙kg^−1^, and wheat exposed to bacteria 4CH and herbicide 0.25 mg∙kg^−1^.

Shoots (*n* = 30) aged 18 days were weighed immediately after cutting using analytical scales HR-250AZG (AND, Tokyo, Japan). Biochemical assays were conducted on the 14–17th day after seedling emergence.

### 2.3. Non-Enzymatic Antioxidants

L-ascorbic acid was determined by titration with DCPIP (2,6-dichlorophenolindophenol) as described by Dewhirst et al. [27]. Leaves (1 g) were ground in 5 mL of 2% (*w*/*v*) metaphosphoric acid and centrifuged at 5000g for 10 min. An amount of 1 mL supernatant was titrated with 3.73 mM 2,6-dichlorophenolindophenol (DCPIP). The volume of DCPIP added was compared to a standard curve of ascorbic acid concentrations. Reduced glutathione was assayed by an enzyme recycling procedure [28] in which it was sequentially oxidized by 5,5′-dithiobis-2-nitrobenzoic acid (DTNB) and reduced by NADPH in the presence of GR. The number of flavonoids in leaves was evaluated using the DUALEX SCIENTIFIC+ device (FORCE-A, Orsay, France) in accordance with the manufacturer’s recommendations. The method is based on the ability of certain flavonoids to fluoresce. The measurements were conducted on five independently prepared samples from each group of plants.

### 2.4. Malondialdehyde Assay

The amount of malondialdehyde (MDA) was measured by thiobarbituric acid-reactive-substances assay, improved by Hodges et al. [29]. According to it, absorbances were read at 440 nm, 532 nm, and 600 nm. The absorbance at 532 nm of a solution containing plant extract incubated without TBA from an identical solution containing TBA was subtracted. The amount of MDA was calculated using the formula proposed by the authors. The measurements were conducted on five independently prepared samples from each group of plants.

### 2.5. Antioxidant Enzyme Assays in Crude Leaf Extract

The leaves were cut from several plants and randomly divided into three samples. Then, 500 mg of fresh leaf tissue was collected from each sample and ground to a fine powder in liquid nitrogen using a precooled mortar and pestle. Each powdered sample was thoroughly homogenized in 5 mL of 0.1 M potassium phosphate buffer (pH 7.4 with 0.1 mM EDTA). The samples were centrifuged at 5000× *g* for 20 min at 4 °C. The clear supernatant was decanted, stored at 4 °C, and used twice for analyses for an hour. Therefore, every value was the average of six repetitions.

Glutathione reductase (GR) activity was assayed according to Smith et al. [30]. GR is a NADPH-dependent enzyme that catalyzes the reduction of oxidized glutathione (GSSG) to reduced glutathione (GSH). The activity of GR is measured by following the reduction of 5,5-dithio-bis-(2-nitrobenzoic acid) (DTNB) to 2-nitro-5-thiobenzoic acid (TNB) by GSH. The increase in absorbance per unit time at 412 nm due to the formation of TNB is determined using a spectrophotometer. The reaction mixture consisted of 1.5 mL of 0.05 M potassium phosphate buffer (pH 7.8) with 2 mM EDTA, 0.15 mL of 20 mM oxidized glutathione (GSSG), 0.15 mL of 2 mM NADPH, 1 mL of 3 mM DTNB, and 0.2 mL of leaf extract. GSSG was added last to initiate the reaction, and the increase in absorbance was recorded for 3 min. The extinction coefficient of TNB (14.15 M^−1^ cm^−1^) was used to calculate the activity of GR that was expressed in terms of units (millimole TNB) per minute per gram fresh weight.

Superoxide dismutase (SOD) activity was assayed using a modified NBT method [31,32]. SODs are metalloproteins that catalyze the dismutation reaction of two superoxide free radicals to one molecule of O_2_ and one molecule of H_2_O_2_. Nitroblue tetrazolium (NBT) is reduced to monoformazon by the superoxide radical. SOD activity is quantified based on the competitive inhibition of NBT reduction by the superoxide radical. The 3 mL assay reaction mixture contained 50 mM phosphate buffer (pH 7.8) containing 2 mM EDTA, 10 mM L-methionine, 0.055 mM NBT, and 0.025% Triton-X100. Then, 0.1 mL of the sample and 50 μL of 1 mM riboflavin were added, and the reaction was initiated by illuminating the samples under a 15 W fluorescent tube. During the 10 min exposure, the test tubes were placed in a box lined with aluminum foil. Duplicate tubes with the same reaction mixture were kept in the dark and used as blanks. The absorbance of the samples was measured immediately after the reaction was stopped at 560 nm. The activity of SOD was expressed in terms of units per minute per gram of fresh weight.

Ascorbate peroxidase (APX) activity was assayed using a modified method of Nakano and Asada [32,33]. APX activity was determined from the decrease in absorbance at 290 nm due to the oxidation of ascorbate in the reaction. The 3 mL assay mixture contained 50 mM potassium phosphate buffer (pH 7.0), 0.5 mM ascorbate, 0.5 mM H_2_O_2_, and 0.1 mL of crude leaf extract. H_2_O_2_ was added last to initiate the reaction, and the decrease in absorbance was recorded for 3 min. The extinction coefficient of 2.8 mM^−1^ cm^−1^ for reduced ascorbate was used in calculating the enzyme activity that was expressed in terms of units (millimole of ascorbate) per minute per gram fresh weight.

CAT activity was determined as described [32]. The decomposition of H_2_O_2_ was followed by a decrease in absorbance at 240 nm. The 3 mL assay mixture contained 50 mM potassium phosphate buffer, pH 7.0, 10 mM H_2_O_2_, and 0.1 mL leaf extract. The extinction coefficient of H_2_O_2_ (40 mM^−1^ cm^−1^ at 240 nm) was used to calculate the enzyme activity that was expressed in terms of units (millimoles of H_2_O_2_) per minute per gram of fresh weight.

### 2.6. In Vivo Activity of ALS

The in vivo assay ALS activity was represented by the accumulation of the substrate. It can monitor the activity inhibition by the ALS-inhibiting herbicide that is absorbed and even partially degraded by plants. Simpson et al. ’s [34] and Liu et al. ’s [24] descriptions of the assay were used. In brief, the plants were sprayed with 0.5 mol/L of 1,1-cyclopropanedicarboxylate (CPCA), a specific inhibitor of ketol-acid reductoisomerase (KARI) subsequent to ALS, to accumulate ALS enzyme substrate acetolactate. After 24 h, leaf samples were collected from the CPCA-treated plants, and 1 g sample was extracted with 5 mL of 0.05 M sodium phosphate buffer (pH 7.2) and centrifuged for 10 min at 5000× *g*. An amount of 0.5 mL supernatant was incubated at 60 °C for 10 min. Then, 0.1 mL of 1 M sulfuric acid was added to stop the catalysis of the ALS enzyme, and the incubation was maintained for 30 min to generate acetoin. Then, 1 mL of 0.5% (*w*/*v*) creatine (prepared in 2 M NaOH) and 1 mL of 5% (*w*/*v*) alpha naphthol (in 2 M NaOH) were added and kept at 37 °C for 30 min to transform acetoin into a red compound. The supernatant was obtained after instant centrifugation and detected by using a spectrophotometer. The absorbance of the supernatant was measured at 530 nm (A530), and then ALS activity was calculated as A530 per hour by 1 g fresh sample. Each sample had six replicates.

### 2.7. Statistical Analysis

The “random samples” were generated by Microsoft Excel 2010 and used for sampling plants. The data were analyzed using the Statistica program (Statsoft) (version 10). The significance of differences between mean values was assessed by ANOVA followed by Duncan’s test (*p* ≤ 0.05).

## 3. Results

### 3.1. Plant Weight

**Figure 2:**
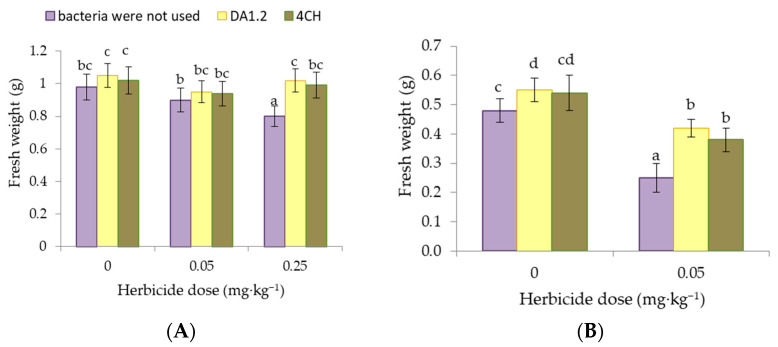
Effect of strains *Pseudomonas protegens* DA1.2, *P. chlororaphis* 4CH, and metsulfuron-methyl treatment on the fresh weight (FW) of one wheat (**A**) or rapeseed (**B**) shoot; significantly different means are indicated by different letters (*p* < 0.05, *n* = 30).

The results of the weight of wheat and canola plants exposed to different concentrations of metsulfuron-metil are presented in Figure 2. In our experience, the effect of a dose of 0.05 mg∙kg^−^^1^ of metsulfuron-methyl on the weight of wheat plants was not significant (*p* > 0.05). While a dose of 0.25 mg∙kg^−^^1^ worsened wheat growth immediately after application, the shoots weighed less by 14.0%. The weight of one wheat shoot after treatment with *P. protegens* DA1.2 was 27.5% higher compared to the analogs without it when using a herbicide at a dose of 0.25 mg∙kg^−^^1^. Under the same conditions, the spraying of wheat with bacterial culture *P. chlororaphis* 4CH provided a 23.8% increase in shoot weight. A similar trend was observed for the weight of canola shoots. After the application of metsulfuron-methyl, the soil significantly depressed the development of canola plants. The mass of one plant exposed to the chemical decreased, compared to the non-herbicidal control, by 47.9%. The formation of the first leaves slowed down. The positive effect of the strains *P. protegens* DA1.2 and *P. chlororaphis* 4CH was established both in conditions favorable for canola and against the background of herbicide. After bacterial treatment, the weight of the shoots was 87.5% and 79.2% of the control values if metsulfuron-metil was applied. It increased to 114.6%, and 112.5% of the control values herbicide was not used.

### 3.2. Lipid Peroxidation

**Figure 3:**
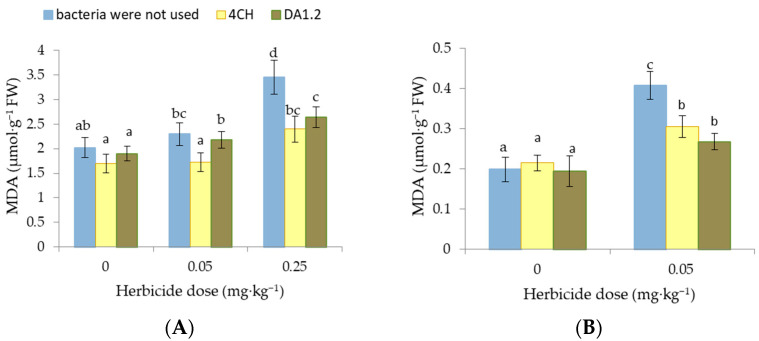
Effect of *Pseudomonas protegens* DA1.2, *P. chlororaphis* 4CH, and metsulfuron-methyl treatment on the MDA level in wheat (**A**) and rapeseed (**B**) shoots; significantly different means are indicated by different letters (*p* < 0.05, *n* = 5).

As an indicator of lipid peroxidation in experimental plants, the content of MDA in the leaves was chosen. The amount of MDA in the leaves of control wheat plants was 2.02 μmol∙g^−^^1^. It remained at this level in all samples excluding wheat leaves exposed to 0.25 mg∙kg^−^^1^ of metsulfuron-metil alone and a combination of herbicide and bacterial culture *P. protegens* DA1.2 (Figure 3). In these samples, the amount of MDA was 70.8% and 30.7% higher than the control value. When soil was contaminated with herbicide residues, the MDA amount in canola leaves increased by 105.6% (*p* < 0.01), indicating oxidative damage. In contrast, the use of bacteria led to the inhibition of the MDA formation in the canola leaves. Due to inoculation by strains *P. protegens* DA1.2 and *P. chlororaphis* 4CH, the amount of MDA in the plants from herbicide-contaminated pots decreased by 34.4% (*p* < 0.05) and 25.2% (*p* < 0.05), respectively.

### 3.3. Non-Enzymatic Antioxidants

The amount of ascorbic acid varied in the samples of wheat and rapeseed leaves (Table 2 and Table 3). In some samples, it exceeded the control values. Examples of this were wheat plants exposed to 0.05 mg∙kg^−^^1^ of metsulfuron-methyl. On the contrary, the canola plants exposed to herbicidal residues and strain DA1.2 contained less ascorbic acid than in the control. This was also true for wheat samples exposed to 0.25 mg∙kg^−1^ of metsulfuron-methyl or its combination with the strain DA1.2. However, we were unable to identify any patterns in the data we received.

There were differences in the amount of glutathione between plant groups depending on the use of the herbicide, bacterial cultures, and the species of the test plants. Compared with wheat, rapeseed accumulated glutathione more actively in response to herbicidal soil contamination. In rapeseed shoots from herbicide-contaminated pots, the amount of GSH was 25.0% higher than in the control. In the case of adding bacterium DA1.2 or 4CH to these pots, it was 285.5% or 85.6% higher than control values. As for wheat, the concentration of GSH fluctuated in the samples to a lesser extent. Noticeable differences in its amount were found between the control group and the group exposed to 0.05 mg∙kg^−^^1^ of herbicide (*p* < 0.05), as well as groups exposed to both doses of herbicide and groups exposed to a combination of herbicide and bacteria (*p* < 0.05). Thus, in our experiment, bacterial cultures DA1.2 and 4CH contributed to the accumulation of GSH in wheat and canola shoots.

In wheat and canola leaves, the flavonoid content changed in a similar way after the application of bacterial strains or metsulfuron-methyl. The presence of metsulfuron-metil in a dose of 0.25 mg∙kg^−^^1^ provoked the accumulation of flavonoids in wheat leaves by 25.7%. If, in addition to the herbicide, bacterial cultures were used, then there was a tendency to increase this percentage. Therefore, under 0.25 mg∙kg^−^^1^ of metsulfuron-methyl, a significant difference (*p* < 0.05) was shown between plants exposed to strains DA1.2 or 4CH and plants not exposed to them, but not between two bacterial strains. The base level of flavonoids in the leaves of the canola plants was 0.185 units. In plants grown in soil containing herbicide residues, the level of flavonoids was 42.7% higher, and treatment with bacteria enhanced this effect. 

### 3.4. Antioxidant Enzymes

**Figure 4:**
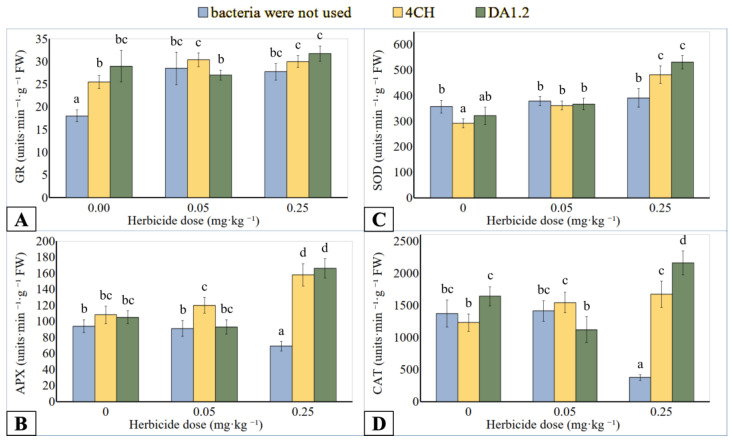
Effect of *Pseudomonas protegens* DA1.2, *P. chlororaphis* 4CH, and metsulfuron-methyl treatment on the activity of GR (**A**), APX (**B**), SOD (**C**), and CAT (**D**) from wheat shoots; significantly different means are indicated by different letters (*p* < 0.05, *n* = 6).

**Figure 5:**
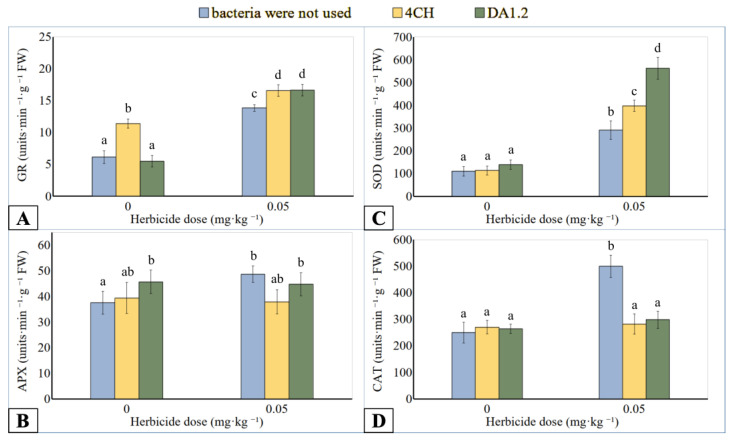
Effect of *Pseudomonas protegens* DA1.2, *P. chlororaphis* 4CH, and metsulfuron-methyl treatment on the activity of GR (**A**), APX (**B**), SOD (**C**), and CAT (**D**) from canola shoots; significantly different means are indicated by different letters (*p* < 0.05, *n* = 6).

GR catalyzes the reduction of oxidized glutathione with the participation of NADPH(H+) in the glutathione-ascorbate cycle. GR activity of leaf extracts increased up to 50.0–76.4% (Figure 4A) or 125.0–170.7% (Figure 5A) if wheat or canola plants were in contact with metsulfuron-methyl. No significant differences in enzyme activity were recorded when wheat was exposed to herbicide doses of 0.05 mg∙kg^−^^1^ and 0.25 mg∙kg^1−^. In addition, the effect of treatment on GR activity was not significant after the joint application of bacteria DA12 and 4CH and herbicide compared to using only metsulfuron-methyl. However, the tendency to higher GR activity after the use of bacteria in wheat variants with a herbicide dose of 0.25 mg∙kg^−^^1^ and canola variants was observed. However, the effect of bacteria alone on activity was contradictory. The enzyme from the leaves of wheat treated with bacteria DA12 and 4CH and canola leaves treated with a bacterial strain 4CH was more active than the control one. The increase was 60.5%, 41.7%, and 85.0%, respectively. At the same time, the treatment of canola with a bacterial strain DA12 did not affect the activity of this enzyme.

CAT catalyzes H_2_O_2_ into water and oxygen in the plant cells. In the AA-GSH pathway, APX plays a central role in the scavenging of H_2_O_2_ and utilizes ascorbic acid as an electron donor. The use of metsulfuron-methyl in an amount of 0.25 mg∙kg^−^^1^ inhibited the APX activity in wheat samples up to 26.6% (Figure 4B), while CAT activity up to 72.6% (Figure 4D) was less than the reference value. Bacterial treatment induced increased activity of APX and CAT extracted from the wheat leaves if the metsulfuron-methyl dose was 0.25 mg∙kg^−^^1^ of soil. Compared to herbicide application without bacterization, the APX activity of *P. protegens* DA12 and *P. chlororaphis* 4CH treated plants was increased up to 140.6% and 129.0%, respectively. A similar comparison showed an increase in the activity of CAT from DA12- and 4CH-treated plants by 476% and 345%, respectively. The differences in enzyme activity between the other wheat plants were not significant regardless of the use of herbicide or bacteria. On the contrary, the APX activity of the canola exposed to metsulfuron-methyl was 29.9% higher than that of control plants (Figure 5B). After treatment with bacterial strain DA1.2, the APX activity of the canola samples tended towards the level achieved using only herbicide. The canola CAT activity was significantly stimulated by the herbicide residues and was reduced after DA12 and 4CH use by up to 40.4% and 46.0%, respectively (Figure 5D).

SOD catalyzes the reduction reaction of the superoxide radical to hydrogen peroxide. Analogies were observed in the change in the activity of SOD, CAT, and APX of wheat samples after treatment with bacteria or metsulfuron-methyl. The SOD activity in wheat leaf extract was higher than control only under the combined effect of a high dose (0.25 mg∙kg^−^^1^) of herbicide and bacterial treatment (Figure 4C). If a culture of bacterium *P. protegens* DA12 was used for spraying wheat, the activity of SOD increased by 48.7%. If a culture of bacterium *P. chlororaphis* 4CH was applied, the activity of SOD increased by 35.0%. After contact of canola plants with metsulfuron-methyl residues in the soil, the activity of SOD in them increased by 164.5% (Figure 5C). The treatment of canola with bacterial cultures against the background of herbicidal soil contamination was accompanied by an increase in SOD activity compared to using only herbicide. For the bacterial strain DA1.2, the increase was 93.5%, and for the bacterial strain 4CH, it was 36.8%. 

### 3.5. ALS Activity

**Figure 6:**
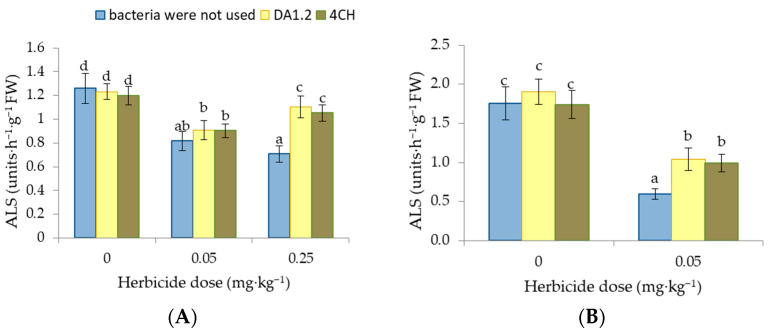
Effect of strains *Pseudomonas protegens* DA1.2, *P. chlororaphis* 4CH, and metsulfuron-methyl treatment on the ALS activity in wheat (**A**) and rapeseed (**B**) shoots; significantly different means are indicated by different letters (*p* < 0.05, *n* = 6).

The effect of metsulfuron-methyl on canola and wheat plants resulted in the inhibition of ALS activity. In wheat leaves, the enzyme activity decreased by 35% and 44% under the influence of 0.05 mg∙kg^−^^1^ and 0.25 mg∙kg^−^^1^ of metsulfuron-methyl, respectively (Figure 6). The inhibition of ALS caused by metsulfuron-methyl was compensated to a greater or lesser extent after the treatment of wheat and canola with cultures of growth-stimulating bacteria. Bacterization with *P. protegens* DA1.2 and *P. chlororaphis* 4CH restored the activity of ALS in wheat leaves to 84% and 88% of the control level if the herbicide dose was 0.25 mg∙kg^−^^1^. If we take into account only pots with this dose of herbicide, the samples with and without growth-stimulating bacteria differed significantly (*p* < 0.01). In rapeseed plants, after the use of bacterial strains *P. protegens* DA1.2 and *P. chlororaphis* 4CH, ALS activity increased to 59% and 50% of the control level.

## 4. Discussion

Dose-dependent inhibition of wheat growth was found when the plants were exposed to different concentrations of metsulfuron-methyl. This is evidenced by the lower weight of the wheat plants after application of the herbicide at a dose of 0.25 mg∙kg^−1^ compared to a dose of 0.05 mg∙kg^−1^. As expected, metsulfuron-methyl was much more toxic to canola than to wheat. Even 4 months after soil contamination, herbicide residues greatly reduced the weight of canola shoots. Next, we will focus on similar trends for wheat and rapeseed crops. Discrepancies in enzyme activity and amount of metabolites between rapeseed and wheat plants could be caused by several reasons (species characteristics, sensitivity to herbicide, or differences in treatment). The design of the experiments does not allow us to assess the significance of these factors.

In sensitive plants, the enzyme ALS is the site of action of metsulfuron-methyl. The resulting essential aliphatic amino acids and protein deficiency are accompanied by various secondary effects of ALS inhibition such as depletion of intermediates of the pathway for some critical processes and disruption of photosynthesis, transport, and respiration systems [35]. Eceiza et al. [36] have shown that oxidative stress is linked to ALS inhibition. This explains the phytotoxicity that we observed. Photosynthesis or respiration disorder may be associated with oxidative stress and the accumulation of MDA in the leaves of rapeseed and wheat as a manifestation of the toxicity of metsulfuron-methyl. The MDA is formed as a result of lipid peroxidation, so its amount in cells correlates well with the content of oxidants and oxidative stress levels [37]. Based on our experimental data, we can conclude that exceeding the dose of metsulfuron-methyl caused oxidative stress in wheat. Signs of oxidative stress were also observed in rapeseed plants grown in soil containing herbicidal residues.

In our experiment, bacterial stimulants *P. protegens* DA1.2 and *P. chlororaphis* 4CH acted as a safener for canola and wheat from the phytotoxicity of metsulfuron-methyl. Spraying with bacteria led to a decrease in the amount of MDA in wheat and canola leaves. For canola, against the background of metsulfuron-methyl, this effect was shown for the first time. However, it was previously observed when combining other crops with herbicides of a different chemical nature [16,17,18,19,20]. The study of Ibrahim et al. [38] concluded that foliar spray of pongamia oil alleviated the toxic effects of glyphosate on tomato plants in the form of increased root and shoot biomass while reducing MDA and O2. A similar pattern was observed in our previous research on sugar beet [39], where the strain DA 1.2 of *P. protegens* reduced the amount of MDA in the leaves when herbicide was applied. 

The ALS activity of canola as a sensitive crop was strongly suppressed by metsulfuron-methyl, and the ALS activity of wheat as an insensitive culture was reduced only by an exceeded dose of herbicide. The effect of sulfosulfuron on the concentration of amino acids was demonstrated even in wheat, which is weakly sensitive to ALS inhibitors [40]. The decrease of ALS products caused by metsulfuron-methyl was compensated to a greater or lesser extent after the treatment with cultures of growth-stimulating bacteria *P. protegens* DA1.2 or *P. chlororaphis* 4CH. We have not found articles that explain this effect. Nevertheless, we can assume that the bacteria destroyed metsulfuron-methyl or enhanced the detoxification of the herbicide by plants. The biodegradation or biotransformation of herbicides by both microorganisms and plants has been demonstrated by researchers [41,42]. The influence of interactions between plants and microorganisms on these processes still requires detailed study. 

Antioxidant protection of plants is implemented through a multicomponent system of enzymes, non-enzyme antioxidants, and signaling molecules. In plants, the ascorbate–glutathione pathway plays a central role in combating the wrong-doings of reactive oxygen species. It includes both nonenzymatic (ascorbate, glutathione) and enzymatic components (APX and GR) [43,44]. Our results indicated differences in the reaction of some antioxidant compounds to the treatment of plants by bacteria against the background of herbicidal stress. GSH is a low molecular weight tripeptide, which is a strong antioxidant and an essential metabolite with a multifarious role in plants. Our study showed the accumulation of GSH in the leaves of rapeseed and wheat as one of the reproducible reactions of these plants to the simultaneous treatment of metsulfuron-methyl and bacteria. Several works not related to herbicides also indicate that growth-stimulating bacteria can contribute to the accumulation of GSH by plants. For example, two PGPR strains belonging to the genus *Pantoea* and *Enterococcus* increased GSH content in mash bean plants [45]. *Kocuria rhizophila* enhanced the production of GSH as well as GSSG in the maize plants grown [46]. In both studies, the plants were exposed to salt stress. In plants exposed to herbicides, the role of GSH is not only limited to preventing oxidative damage. Glutathione conjugates with xenobiotics spontaneously or is catalyzed by glutathione transferase to inactivate them. It is assumed that this process is necessary for the transport of the metabolite into the vacuole and possible further decomposition inside the vacuole [22]. In addition, conjugates of glutathione and hydrophobic compounds become more soluble and less toxic. The compounds targeted in this way by glutathione and glutathione transferases cover a wide range of toxins of natural and anthropogenic origin, including drugs, pesticides, and herbicides [47,48]. Apparently, the above applies to metsulfuron-methyl. Amador et al. [49] demonstrated molecular docking of metsulfuron and the glutathione tripeptide, and it represents a possible in silico evidence of glutathione conjugation with this herbicide. In connection with the above, glutathione-metsulfuron-metil conjugates seem one possible explanation for how bacteria improve the condition of plants under herbicidal stress.

Glutathione reductase is a NADPH-dependent enzyme that catalyzes the reduction of oxidized glutathione (GSSG) to reduced glutathione (GSH). GR helps to maintain a high ratio of GSH/GSSG as part of the ascorbate–glutathione cycle. Therefore, the increase in GR activity registered by us simultaneously with an increase in the amount of GSH seems natural. However, previous studies show both activation and inhibition of the enzyme GR in plants under the influence of plant growth-promoting bacteria and various stresses. For example, inoculated plants showed increased activities for several antioxidant enzymes (catalase, peroxidase, polyphenoloxidase, and glutathione reductase) when ryegrass was used for amelioration of mine tailings [50]. On the other hand, inoculation with two PGPR strains, *Serratia* sp. and *Rhizobium* sp., into saline soils decreased APX and GR activity [51]. Apparently, this reflects the sensitivity of GR to the type of stress. Maintaining the level of GSH/GSSG is important for herbicide detoxification, so the bacteria contributing to this had an anti-stress effect.

It is of interest to compare the results of our work with the conclusions of studies of chemical safeners. As the bacterial treatment used in our study, some safeners prevented reductions in ALS activity caused by herbicides. Naphthalic anhydride was reported as the most effective antidote in this respect, preserving ALS activity in corn roots and shoots and in sorghum shoots [52]. The mechanisms of action of safeners presumably include the induction of enzymes that transform herbicide molecules (monooxygenase, glucosyltransferase) or conjugation with glutathione [53,54]. In addition, safeners enhance the transport of glutathione or glucose conjugates with herbicides [55]. An increase in the amount of glutathione in wheat and rapeseed leaves after the use of bacteria may be part of the protective mechanism, as we suggested above. Thus, the assumption that chemical and microbiological antidotes of herbicides can affect biochemical processes in a plant in a similar way deserves consideration and further experimental verification.

Ascorbic acid acts as an antioxidant, directly absorbing reactive oxygen species that are formed during photosynthesis and respiration. Studies have shown that exogenous use of ascorbic acid can alleviate stress caused in plants by herbicide isoproturon [56] and nicosulfuron [57]. Kandoliya and Vakharia [58] showed that *Pseudomonas fluorescens*, a common PGPR, has the ability to elicit an antioxidant system including ascorbic acid. In a study conducted on oxidative stress tolerance developed in wheat under Zn stress, Islam et al. [59] found that *Pseudomonas aeruginosa* enhanced the production of ascorbic acid to combat the adverse effects of Zn stress. However, in the leaves of wheat and rapeseed treated with metsulfuron-methyl, the amount of ascorbic acid and APX activity increased only in some variants of the experiment. In other experimental groups, the level of ascorbic acid even decreased, especially after the use of the bacterium DA1.2. This can be explained both by its active consumption in redox reactions or by the weak response of the ascorbate pathway to the treatments we used. The CAT activity also reacted inconsistently to the application of bacterial strains. Bacterial treatment rather mitigated changes in the activity of the enzyme provoked by the herbicide. Among the numerous studies of catalase and peroxidase activity under stressful conditions, conclusions about the activation of these enzymes after the use of growth-stimulating bacteria prevail. Such results were obtained for canola under salinity [60,61], Cd stress [62], and wheat under Zn stress [59], drought [63,64], and salinity [65]. However, our data indicated that the changes in CAT activity were a consequence rather than a cause of the alleviation of herbicidal stress.

The reaction of the SOD to the simultaneous treatment of plants with herbicides and growth-stimulating bacteria has been little studied. Jiang et al. [20] investigated the effects of strain *Pseudomonas chlororaphis* PAS18 and reported enhancing SOD activity in plants. According to Iwaniuk et al. [66], sulfosulfuron or its combination with biostimulants induced the activity of SOD and CAT in wheat. In our study, treatment with bacteria led to an increase in the activity of SOD in herbicide-stressed plants. On the other hand, bacteria did not contribute to the growth of SOD activity if they were not combined with herbicidal treatment. This may indicate the role of this enzyme in mitigating herbicidal stress and improving the growth of bacteria-treated plants. This is consistent with the results of the bacterization of plants suppressed by salinization, drought, and other stress factors [67].

Plant flavonoids are considered substances with antioxidant functions, although there is still a discussion about their protective role in different cellular compartments [68]. We observed the increasing flavonoid amounts in canola and wheat leaves in response to the metsulfuron-methyl and bacteria use. Growth-stimulating microorganisms may affect the biosynthesis of flavonoids in crops. Chamam et al. [69] demonstrated that phenolic compounds such as flavonoids and hydroxycinnamic derivatives were the main rice metabolites affected in response to *Azospirillum* bacteria. Nasab et al. [70] found an accumulation of flavonoids in the canola plants after applying growth-promoting rhizobacteria *Pseudomonas fluorescens, Azospirillum brasilense*, and *Azotobacter chroococcum.* The accumulation of flavonoids may be associated with plant protection against herbicides. Cummins et al. [71] associate multiple herbicide resistance of black grass with an inducible activation in antioxidant and secondary metabolism including elevated levels of flavonoids.

Based on the data of our study, we can conclude that the treatment with bacterial cultures rather stimulated the antioxidant protection of canola and wheat plants. However, the causal relationship between stress alleviation and antioxidant status is still unclear. The enhancement of antioxidant protection may improve the detoxification of metsulfuron-methyl and ALS activity, or the recovery of ALS activity may provide antioxidant reactions with the necessary enzymes and substrates. At least the activity of ALS under the influence of bacterial treatment was not restored enough to completely prevent oxidative stress. As previously reported, resistant plants that contain mutated ALS do not suffer oxidative damage nor do they increase their antioxidant activity [36,72].

## 5. Conclusions

To sum up, the protective role of strains *P. protegens* DA1.2 and *P. chlororaphis* 4CH for wheat and canola plants in response to herbicide stress may be linked to the fact that bacterial treatment can increase the activity of antioxidant enzymes and the amount of glutathione. Most often, the enzymes GR and SOD reacted to herbicidal and bacterial treatment. This can partly explain the reduction in oxidative damage and phytotoxicity that we have observed. Furthermore, the use of bacterial strains DA1.2 and 4CH has a positive effect on ALS activity, which indicates the need for further research on how plant growth-stimulating bacteria affect the inactivation and degradation of metsulfuron-methyl in plants and soil. Since the treatment of plants was carried out by two taxonomically similar strains in the greenhouse, in the future it is planned to increase the diversity of tested bacteria, and field trials will be needed to check the beneficial effect of bacterial treatment on the antioxidant status and ALS activity of crops under the actual use of herbicides in crop rotation.

## Figures and Tables

**Table 1:** Properties of bacterial strains *Pseudomonas protegens* DA1.2 and *Pseudomonas chlororaphis* 4CH [25,26].

Properties	Bacterial Strains	
	*P. protegens* DA1.2	*P. chlororaphis* 4CH
Indole-3-acetic acid production, mg/L	0.870	0.837
P solubilization	capable	capable
Nitrogenase activity, nmol C_2_H_4_ h^−1^ mL^−1^	21.3	30.5
Phytopathogenic fungi suppressed by bacteria	*Alternaria alternata* (Fr.) Keissl., *A. solani* Sorauer, *Bipolaris sorokiniana* (Sacc.) Shoemaker, *Botrytis cinerea* Persoon, *Fusarium culmorum* (W.G. Smith) Sacc., *F. gibbosum* Appel et Wollenw, *F. graminearum* Schwabe, *Rhizoctonia solani* J.G. Kuehn.	*A. alternata* (Fr.) Keissl., *A. solani* Sorauer, *B. sorokiniana* (Sacc.) Shoemaker, *Bot. cinerea* Persoon, *F. culmorum* (W.G. Smith) Sacc., *R. solani* J.G. Kuehn.

**Table 2:** Non-enzymatic antioxidants in wheat shoots exposed to herbicide and bacteria.

Bacterium	Herbicide Dose, mg∙kg^−1^	Ascorbic Acid, µg∙g^−1^ FW	GSH, µg∙g^−1^ FW	Flavonoids, Units
Without bacterium	0	532 ± 39 cde *	30.39 ± 2.04 b	0.226 ± 0.019 a
	0.05	667 ± 45 f	20.95 ± 1.78 a	0.237 ± 0.013 a
	0.25	256 ± 37 b	24.55 ± 1.95 a	0.284 ± 0.008 c
*Pseudomonas protegens* DA1.2	0	480 ± 34 c	37.25 ± 2.28 d	0.246 ± 0.007 ab
	0.05	538 ± 28 de	35.69 ± 1.74 cd	0.259 ± 0.009 b
	0.25	126 ± 21 a	41.13 ± 1.90 de	0.301 ± 0.014 d
*P. chlororaphis* 4CH	0	580 ± 30 e	35.62 ± 1.67 cd	0.232 ± 0.008 a
	0.05	460 ± 45 c	33.61 ± 1.41 bc	0.265 ± 0.011 b
	0.25	599 ± 29 ef	42.39 ± 2.33 e	0.324 ± 0.015 d

* For each column, significantly different means are indicated by different letters (*p* < 0.05, *n* = 5).

**Table 3:** Non-enzymatic antioxidants in canola shoots exposed to herbicide and bacteria.

Bacterium	Initial Dose of Herbicide, mg∙kg^−1^	Ascorbic Acid, µg∙g^−1^ FW	GSH, µg∙g^−1^ FW	Flavonoids, Units
Without bacterium	0	401 ± 32 b *	27.63 ± 1.57 a	0.185 ± 0.021 a
	0.05	520 ± 41 c	34.54 ± 1.89 b	0.264 ± 0.007 c
*Pseudomonas protegens* DA1.2	0	434 ± 36 b	30.67 ± 2.11 ab	0.229 ± 0.015 b
	0.05	262 ± 39 a	106.43 ± 4.25 d	0.323 ± 0.015 d
*P. chlororaphis* 4CH	0	453 ± 43 bc	34.82 ± 1.60 b	0.203 ± 0.023 ab
	0.05	478 ± 35 c	51.28 ± 5.05 c	0.309 ± 0.009 d

* For each column, significantly different means are indicated by different letters (*p* < 0.05, *n* = 5).

## Data Availability

Data is contained within the article.
